# Hydrosalpinx Prolapsed into Douglas’ Pouch—Hydrosalpinx Drained through the Uterus—Chronic Salpingitis and Ovaritis

**Published:** 1894-06

**Authors:** Augustin H. Goelet

**Affiliations:** New York


For Texas Medical Journal.

### HYDROSALPINX PROLAPSED INTO DOUGLAS' POUCH.—HYDROSALPINX DRAINED THROUGH THE UTERUS.—CHRONIC SALPINGITIS AND OVARITIS.

BY AUGUSTIN H. GOELET, NEW YORK.

A Clinical Lecture delivered at the West Side German Clinic, New York.

GENTLEMEN—The first case I will show you to-day is one of hydrosalpinx. The right tube is distended to about the size of a hen's egg, and is prolapsed into Douglas' pouch, bending the uterine end upon itself. This produces complete occlusion and prevents drainage into the uterine cavity.

The patient, Mrs. S——, aged 27 years, has been married six years, but has never been pregnant. She has suffered for the past year with severe backache and pain in the right ovarian region, with dysmenorrhœa and a profuse mucous leucorrhœa. The uterus is in good position, being only slightly anteflexed, and the tube and ovary of the other side appear to be normal. We have been able to relieve her pain and backache by means of fine wire faradization, and she has passed one period with comparative comfort, but it is impossible to remove the tube from its abnormal position, though it does not appear to be adherent. If this could be done, and if it could be maintained in good position, it might be possible to overcome the occlusion and drain it into the uterine cavity, and thus accomplish a cure without resorting to an abdominal section. My experience with such cases, however, leads me to the conclusion that an exsection of the tube is the only means of affording her permanent relief. I shall therefore advise operation. If she consents, you will have an opportunity of witnessing the operation and verifying the diagnosis.

In some of these cases, the treatment instituted in this case will afford complete relief of all the symptoms which will be prolonged for a considerable time, so that if the patient refuses to consent to an operation, or there is any other reason why it should not be done, and she is not obliged to work for a living, she can be made quite comfortable. Treatment may be suspended when relief is obtained, but should be recommenced upon a return of the symptoms. I recall a similar case upon which I operated about a year ago. She at first refused operation, and two months' treatment relieved her absolutely of every symptom. She was warned, however, that the relief would not be permanent. She presented herself three months later, stating that she had suffered no inconvenience since discontinuing treatment, except a slight backache during the last week. The local condition was found to be the same, no alteration in the size of the mass being appreciable. A month later, though there had been no recurrence of the symptoms, she consented to the operation on condition that only one ovary would be removed. The operation was entirely successful, and the patient is in excellent health to-day. The tube was not adherent in Douglas' pouch, and the ovary and tube on the other side were normal.

CASE II. The next case is also one of hydrosalpinx in which so far an operation has not been necessary and the patient's general health, as well as the local condition, is improving every day.

The patient, Mrs J., aged 27 years, has been married five years, and has had one child and one miscarriage, since which she has been in poor health. She has been under observation for something like eight months, though she has not come regularly for treatment. As so many of this class of patients do she has frequently suspended treatment for several months when she has experienced relief. When she first came under my observation she was greatly emaciated from constant suffering, but as you see now, her general condition is very fair.

The uterus was at first retroverted and fixed, both ovaries and tubes were dragged out of position, and the left tube was distended to nearly the size of that in the other case I showed you. The whole vaginal vault was exquisitely sensitive and an exact diagnosis could not at first be distinctly made. The treatment first instituted was vaginal faridization. Later, when the sensitiveness had to a great extent been overcome, mild galvanic applications of the negative pole were made to the endometrium, and these were followed each time by vaginal faridization. We were shortly rewarded by observing immediately after the applications a rather free discharge from the uterus and an evident diminution in the size of the distended tube, showing drainage by the natural channel had been established. This occurred after the second or third application to the endometrium. After the uterus had been rendered moveable and it could be replaced, it was supported at first by tampons, and later by a carefully adjusted pessary.

You will observe upon examinution that the uterus now retains a normal position when the support or pessary is removed. The tube is not distended and though not to be considered perfectly noımal it is evidently giving no inconvenince.

I believe that under favorable circumstances this patient can get entirely well, though the outlook at first was not encouraging.

The same result may be brought about likewise when the tube is distended with pus, if there is not actual occlusion of the uterine end of the tube. In the majority of instances of moderate distention, unless the tube and ovary are prolapsed, producing occlusion, I believe the obstruction at the uterine end is due to tumefaction of the mucous membrane, and can be overcome, allowing drainage into the uterine cavity. Where there is actual occlusion, however, immediate steps should be taken to remove it. There is not that danger in temporizing in these cases of moderately distended tubes as some are inclined to make you believe, because the distal end of the tube is sealed by inflammatory action at an early stage, and leakage into the peritoneal cavity is out of the question, unless rupture occur, which is exceedingly improbable. This is perfectly rational, for it would evidently be impossible for the tube to become distended unless the distal end is occluded. If it remained free, there would be constant leakage into the peritoneal cavity, as that end of the tube is larger than the uterine end, and is dependent; hence less liable to become obstructed.

CASE III. The next case is one of chronic salpingitis and ovaritis, which has been under treatment for two weeks.

The patient, Mrs. McE., aged 24 years, has been married four years but has never been pregnant. She had some dysmenorrhœa previous to marriage, and this became very much worse after, and she suffered with constant pelvic pain and backache, very much exaggerated by walking or standing. She had a profuse muco-purulent leucorrhœa when she first presented herself for treatment, but this is now much less, and thinner in character.

The vaginal vault was exquisitely sensitive to pressure on both sides of the uterus. The ovaries could be distinctly palpated, and were found to be enlarged and tender, but the tubes could not be clearly made out, because there was considerable infiltration. The endometrium was in a condition of similar inflammation, which was no doubt the origin of the tubal inflammation.

You will observe on examination all the physical signs I have mentioned, but you see she bears the examination now very well because the tenderness has been overcome by the treatment she has received, which has consisted of moderate negative galvanic applications to the endometrium and faradization of the vagina. The tubes can now be made out because the infiltration has been removed, and are found to be much thickened, as was to have been expected. The patient has been greatly relieved in the short time she has been under treatment. She has comparatively very little pain and can now walk several blocks without inconvenience, a thing that was out of the question before. There is no reason whatever why this patient should not get entirely well. She will get well under the line of treatment which has been instituted and be able to perform her household duties as well as she ever did. The question may be asked, will she become pregnant? That I cannot answer positively, yet I can see no reason why this will not be possible. My experience with these cases convinces me that under favorable conditions they may be cured without resort to operation. If the patient will exercise even ordinary care and the attendant will faithfully and patiently carry out the treatment, their efforts will be successful. You will have the opportunity to observe this case from time to time, to note the method of treatment and the improvement in her condition.

